# Tonic pain reduces autonomic responses and EEG functional connectivity elicited by affective stimuli

**DOI:** 10.1111/psyp.14018

**Published:** 2022-02-06

**Authors:** Guzmán Alba, Jaime Vila, José G. V. Miranda, Pedro Montoya, Miguel A. Muñoz

**Affiliations:** ^1^ Brain, Mind and Behavior Research Center at University of Granada (CIMCYC‐UGR) Spain; ^2^ Institute of Physics, Laboratory of Biosystems Federal University of Bahia Salvador Brazil; ^3^ Research Institute of Health Sciences (IUNICS) University of Balearic Islands Palma Spain

**Keywords:** attention, EEG functional connectivity, emotion, galvanic skin response, heart rate, pain

## Abstract

Most pain studies have focused on only two aspects of pain: the influence of pain on attentional processing and the modulation of pain perception by affective stimuli. However, the influence of tonic pain on the attentional processing of affective stimuli has not been studied. In this study, we investigated the effects of tonic pain on the attentional processing of affective stimuli, focusing on autonomic responses and their relationship with both EEG power and functional connectivity. Forty participants (20 men and 20 women) received tonically painful and nonpainful thermal stimulation while viewing blocks of pleasant, unpleasant, or neutral images. The galvanic skin conductance response (SCR), electrocardiographic activity, and electroencephalographic (EEG) activity in the delta and theta bands were recorded. Participants rated the unpleasantness of the pain at the end of each block. Typical affective SCR and heart rate (HR) patterns were found in the no‐pain condition, but when the pain was delivered, these patterns disappeared. EEG power and functional connectivity results showed that tonic pain affected the delta band in the central region during pleasant and unpleasant image blocks. Our findings suggest that tonic pain captured attentional focus and reduced the cognitive resources available for processing affective stimuli, altering the emotional experience associated with pain.

## Abstract 2

Previous studies with pain patients suggest that pain reduces the attentional processing of affective stimuli. However, these results could be a consequence of affective disorders related to pain diseases. The current article provides new subjective and physiological evidence that tonic pain reduces the attentional processing of affective stimuli in healthy participants.

## 1 INTRODUCTION

Our brain has developed efficient selection mechanisms that can automatically direct attention to stimuli that are relevant to survival (Bradley et al., 2001; Carretié, 2014; Ferrari et al., 2008; Lang et al., 1997). Accordingly, it has been proposed that the orientation of attention is mediated by defensive and appetitive motivational systems that evolved to protect and sustain an individual’s life (Bradley, 2009; Carretié, 2014; Vila et al., 2007). Thus, threatening stimuli activate defensive actions (such as combat or escape), whereas appetitive stimuli activate approach behaviors. Pain is a biologically relevant and vital signal of bodily threat that inherently attracts and demands attention (Crombez et al., 2013; Priebe et al., 2015). Painful stimuli drive a cascade of protective behaviors including (Bradley & Lang, 1994) increased arousal and prioritization of attention to the sources of pain (van Ryckeghem & Crombez, 2018; Vlaeyen & Crombez, 2020). Numerous studies have extensively demonstrated that pain decreases performance in several attentional tasks such as the n‐back, attentional switching, and divided attention tasks (Attridge et al., 2016; Berryman et al., 2014; Keogh et al., 2014). The disruptive effect of pain on attentional performance depends on the features of the painful stimuli and the cognitive task involved. Painful stimuli are more disruptive to attentional performance when the pain is novel, unpredictable, threatening, more intense, and/or of a longer duration (Attridge et al., 2015; Sinke et al., 2015; van Ryckeghem & Crombez, 2018). In contrast, the disruptive effect of pain is decreased when cognitive tasks load the working memory with information unrelated to pain (Legrain et al., 2011) and/or have goals that are motivationally relevant for participants (Verhoeven et al., 2010). In general, pain decreases attentional performance because painful stimuli compete with task‐target stimuli for attentional resources.

Research has also focused on how affective stimuli modulate pain perception. The general assumption is that pleasant stimuli (music, images, or videos) reduce pain, whereas unpleasant stimuli are associated with increased pain (Lee & Uchiyama, 2015; Rhudy et al., 2006; Roy et al., 2012; Williams & Rhudy, 2009, 2012). Physiological measures have provided further evidence for this assumption. For example, the skin conductance response (SCR) and heart rate (HR) acceleration in response to painful electrical shocks increased when participants viewed unpleasant pictures (Rhudy et al., 2006; 2008; Williams & Rhudy, 2009, 2012). In addition, research on the brain has further suggested that the functional connectivity of frontal regions may be related to the influence of emotions on pain processing (Ploner et al., 2011; Roy et al., 2009). However, these effects occurred only when pain intensity was low or when affective stimuli were more unpleasant than painful (Flaten et al., 2016; Rhudy et al., 2008). Moreover, low‐arousal affective stimuli have been found to enhance pain perception, whereas high‐arousal affective stimuli have been found to capture more attentional resources and to modulate pain perception (Rhudy et al., 2008).

Interestingly, most studies have focused on the influence of pain on attentional processing or on the modulation of pain perception by affective stimuli. Much less is known about how pain influences the attentional processing of affective stimuli. Hints toward a possible influence of pain on affective processing come from studies in pain patients. A recent meta‐analysis of the disruptive effects of pain on the emotional Stroop task revealed that patients with chronic pain or acute pain displayed an attentional bias toward sensory pain words (Todd et al., 2018). In contrast, healthy people displayed an attentional bias toward both sensory pain and affective words (Arioli et al., 2021; Feroz et al., 2017; Quan et al., 2020). The attentional deficits toward affective stimuli observed in pain patients, compared with healthy controls, suggest that pain salience either reduces the attentional processing of affective stimuli or is a consequence of affective disorders related to pain syndromes. Investigating the effect of pain on emotion in healthy populations would allow us to understand the attentional deficits in the processing of affective information experienced by pain patients. To our knowledge, only one study experimentally induced pain in healthy participants and measured its impact on the attentional processing of affective stimuli (Wieser et al., 2012). In that study, tonic pain reduced the response of attentional EEG evoked potentials to affective facial expressions (fearful, neutral, and happy). These findings are concordant with the notion that pain demands attentional resources and reduces the attentional processing of affective stimuli. However, this study was limited in the affective stimuli used and physiological responses analyzed.

The aim of the present study was to increase the understanding of the physiological mechanisms underlying the effect of tonic pain on the attentional processing of affective stimuli. Our study is novel in that we investigated how tonic pain affected autonomic responses as well as EEG power and functional connectivity related to the attentional processing of affective images. In this study, healthy participants viewed blocks of affective images with and without experiencing induced pain. The intensity of the pain stimulus was standardized by adjustment for each participant, and the affective images were selected from the International Affective Pictures System (IAPS) based on the valence and arousal dimensions. Autonomic and central responses have been widely studied in regard to motivational attention to affective images (Bradley, 2009; Gable & Poole, 2014). In general, physiological results have demonstrated that relevant and arousing images garnered more attention and elicited higher skin conductance response (SCR) and greater heart rate (HR) decelerations than neutral images (Bradley, 2009). In our study, we expected that tonic pain would alter the typical autonomic pattern found during the attentional processing of affective images. In terms of brain activity, EEG power and functional connectivity studies have revealed that the delta and theta frequencies of frontal, central, parietal, and occipital electrodes play a significant role in the attentional processing of affective images (Balconi et al., 2009b; Güntekin & Başar, 2014, 2016; Klados et al., 2009). Viewing relevant and arousing images generated enhanced delta and theta oscillations compared with viewing neutral images (for a review: Güntekin & Başar, 2014). Thus, we expect that tonic pain will reduce the delta and theta power related to viewing arousing affective images. Studies on EEG functional connectivity revealed that viewing arousing affective images caused both increases and decreases in the delta and theta connectivity in frontocentral, frontoparietal, centroparietal, fronto‐occipital, and centro‐occipital electrode pairs, depending on the study (Alipour et al., 2019; Güntekin et al., 2017; Lee et al., 2020; Miskovic & Schmidt, 2010). Because of these heterogeneous results, we do not have a clear prediction as to the direction of affective network changes upon the delivery of tonic heat pain stimulation. However, in line with previous results (Wieser et al., 2012), we hypothesized that physiological responses would reflect an increase in pain salience and a decrease in the attentional processing of affective images.

## 2 METHOD

### 2.1 Participants

Forty right‐handed students (20 females and 20 males) participated in the study. They were all students at the University of Granada (average age = 19.86 ± 1.839) who received extra credit in return for their participation. Participant exclusion criteria included students who reported chronic pain, cardiovascular problems, ongoing illicit substance use, or mental health problems or those who were undergoing medical or psychological treatment. Additionally, two participants (two males) were excluded from the study because their recordings had too many artifacts to be properly analyzed. The participants were recruited via the information provided in university classrooms. All participants signed informed consent forms to participate in the study, which was approved by the ethics committee of the University of Granada and performed according to the recommendations of the Declaration of Helsinki.

### 2.2 Quantitative sensory testing

Heat stimulation was administered via a computer‐controlled thermode of a 4 × 4 cm Peltier plate and was individually adjusted for each participant. We used the method of limits to assess heat pain tolerance (as the mean of three measures). The temperatures were delivered starting at 37 °C and were increased until participants considered the heat to be unbearable. The sequence of pain assessments was as follows. First, the participants were instructed to keep the index finger of their left hand in contact with the thermode for 5 s. Then, the participants had to rate the unpleasantness of the temperature using a 0–10 visual analog scale (VAS; 0 represented “no unpleasant temperature,” 5 represented “the temperature is starting to become unpleasant,” and 10 represented “the unpleasantness of the temperature is unbearable”). After the prior temperature was rated, the next temperature (1 °C warmer than the previous temperature) was delivered and evaluated. When the participants rated the unpleasantness of the heat stimulus at 10, the procedure was stopped, and the temperature value was recorded. This procedure was repeated three times for each participant, and the average of the three temperature values was recorded as the individual’s heat pain tolerance. The heat pain stimulus for each participant was calculated as 60% of that individual’s heat pain tolerance (i.e., heat pain stimulus temperature = [0.6 × {average heat pain tolerance − 37 °C} + 37 °C]).

### 2.3 Emotional stimuli

Sixty digital images that evoked pleasant, unpleasant, or neutral emotions were chosen from the Spanish validation of the International Affective Picture System (IAPS; Moltó et al., 1999, 2013; Vila et al., 2001). For each of the three emotional conditions, a set of 20 different images was selected. The “pleasant set” included erotic scenes and sports images (IAPS numbers: 4652, 4658, 4668, 4669, 4670, 4672, 4676, 4681, 8178, 8185, 8186, 8193, 8251, 8300, 8341, 8370, 8400, 8490, 8496, and 8499). The “unpleasant set” included images of mutilation and human and animal attacks (IAPS numbers: 1050, 1113, 2811, 3064, 3100, 3170, 3400, 3550, 6212, 6250, 6263, 6313, 6410, 6550, 6560, 6570.1, 9040, 9120, 9187, and 9400). The “neutral set” included images of mushrooms and household objects (5530, 5531, 5532, 5533, 5534, 7001, 7002, 7003, 7004, 7006, 7009, 7010, 7012, 7020, 7025, 7030, 7031, 7032, 7035, and 7040). To control for the effects of arousal, we selected pleasant and unpleasant images with similar arousal ratings but markedly different valence ratings (see Supplementary Table S1). The neutral images selected had intermediate valence and low arousal ratings.

### 2.4 Procedure

The data were compiled from individual sessions that lasted approximately 90 min. On their arrival at the experimental session, the participants received a brief explanation of the study before signing informed consent forms, followed by a short interview to verify their compliance with the inclusion criteria. Then, the participants completed several questionnaires that were used to characterize psychological variables such as state anxiety (State Anxiety Inventory [STAI]; Spielberger et al., 1970), mood state (Positive and Negative Affect Schedule [PANAS]; Watson & Clark, 1999), and handedness (Edinburgh Handedness Inventory; Oldfield, 1971). The means and standard deviations of these psychological variables are provided in Table 1. Then, the participants were moved to a quiet and dimly illuminated room and seated comfortably for the quantitative sensory test (explained in Section 2.3). Subsequently, SCR, ECG, and EEG electrodes were applied, and the experimental session started.

**TABLE 1:** Means and standard deviations of participants’ psychological data by sex

Questionaries	Males (*n* = 18)	Females (*n* = 20)	Total (*n* = 38)
EHI (mean, SD)	69.15 (18.04)	74.74 (17.32)	72.09 (17.65)
STAI (mean, SD)	11.78 (8.50)	12.35 (5.55)	12.08 (7)
PANAS‐positive (mean, SD)	36.67 (5.32)	36.1 (4.13)	36.37 (4.66)
PANAS‐negative (mean, SD)	20.33 (5.64)	22.55 (4.71)	21.50 (5.13)

**FIGURE 1:**
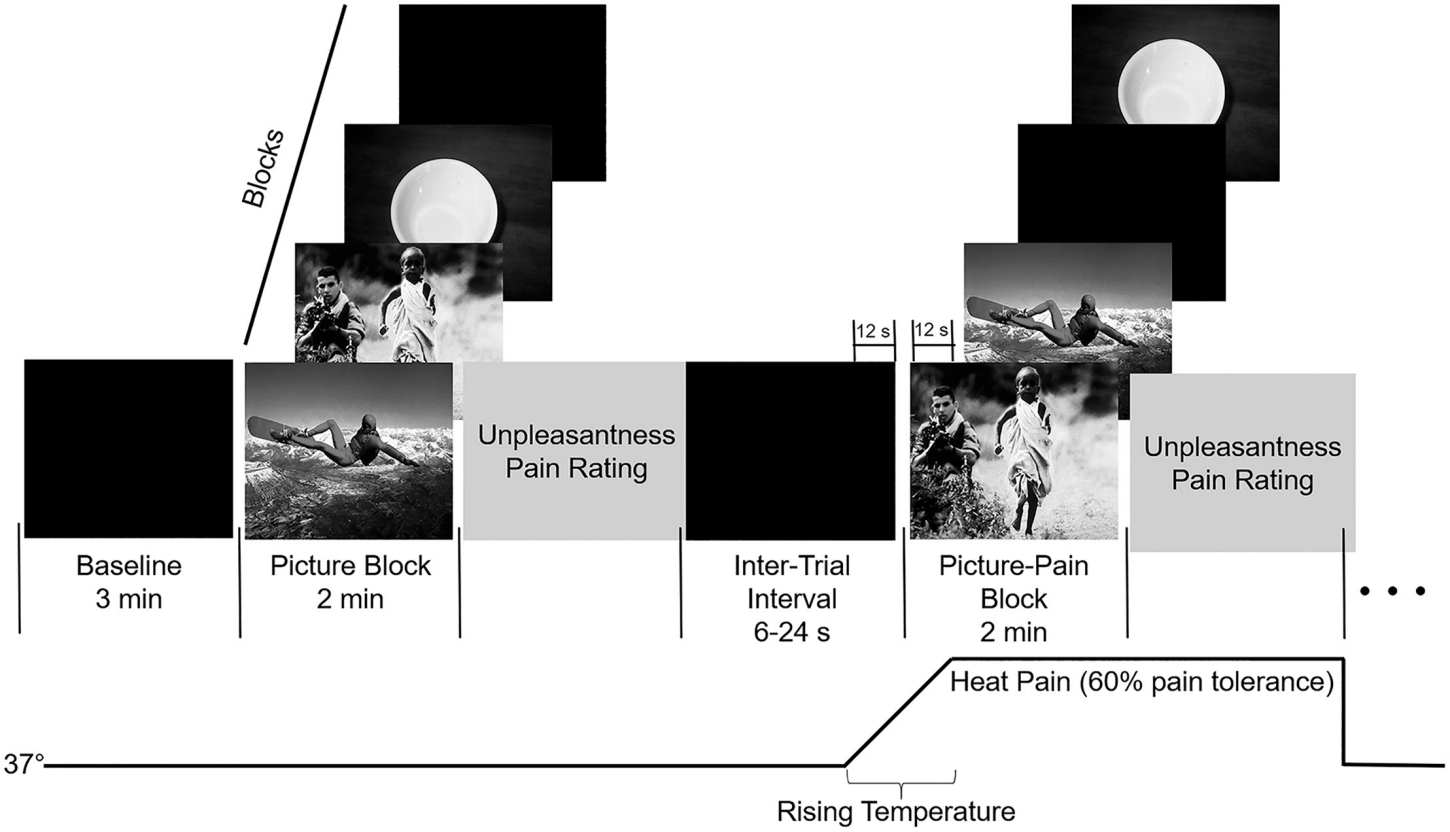
Experimental procedure. A baseline period of 3 min occurred before the task period. During the task, participants viewed three affective blocks (pleasant, unpleasant, and neutral) and one black screen block, each lasting for 2 min. Pain stimuli were delivered in half of the blocks, 12 s after blocks started. Each block was followed by one pain unpleasantness rating and had an ITI of 6–24 s. This entire procedure was repeated after 5 min of rest

Before the experimental task started, the participants were instructed to keep the index finger of their left hand in contact with the thermode at all times and to pay attention to the images that were going to appear on the wall in front of them. The images were projected 3 m in front of the participant with a Canon LV‐53 projector at a size of 140 cm × 95 cm. The task consisted of two 3‐min baseline blocks and eight 2‐min affective blocks (pleasant, unpleasant/pain, neutral, black screen/pain, unpleasant, pleasant/pain, black screen, and neutral/pain) (see Figure 1). The presentation order of the affective blocks was counterbalanced. The task was divided into two parts with a baseline and four different affective blocks: two without pain and two with pain. The black screen condition was subsequently excluded from statistical analyses because the neutral block was used as the control condition. There was a 5‐min rest period between baseline and affective blocks. Pleasant, unpleasant, and neutral blocks contained 20 images from the IAPS that were each continuously presented for 6 s. The intertrial interval (ITI) between blocks was random, oscillating from 6 s to 24 s, and was displayed as a black screen. The pain stimulus was delivered after a risetime of 24 s during which the temperature increased from 37 °C to the individual’s previously calculated heat pain temperature to avoid startle effects. The ramp‐up in temperature started in the last 12 s of the ITI and reached the heat pain temperature in the first 12 s of the pain block. At the end of each affective block, the participants evaluated the temperature’s unpleasantness on the 0–10 VAS. The heat pain stimulus was presented for 1 min and 48 s during the blocks, then the VAS was rated and the temperature had returned to baseline (37 °C). After the completion of the task, the participants evaluated the emotional dimensions (valence and arousal) of each picture using a computerized version of the Self‐Assessment Manikin scale (SAM; Bradley & Lang, 1994).

### 2.5 Physiological data acquisition and preprocessing

Physiological signals from ECG and SCR electrodes were continuously acquired using PowerLab 4/25 T equipment (ADInstruments, Sidney, Australia) and LabChart 5 software (ADInstruments, Sidney, Australia). The electroencephalogram was continuously acquired using the Geodesic Sensor Net connected to a DC‐coupled amplifier (Net Amp 400, Electrical Geodesics, Oregon, United States) and Net Station 4.5 software (Electrical Geodesics, Oregon, United States). The sampling rate of all physiological signals was a frequency of 1,000 Hz.

ECG raw signals were recorded using the lead I configuration (positive and negative sensors placed on the collarbones; ground sensor placed on the right ankle) and filtered with a 1‐ to 35‐Hz bandpass filter. The cardiac period (i.e., the R–R interval) was measured in milliseconds and visually inspected and corrected using an ECG beat detection software program (de Carvalho et al., 2002). Subsequently, KARDIA software was used to transform the cardiac period into a heart rate (HR) measurement in beats per minute (Perakakis et al., 2010). Finally, for each trial, the weighted average of the HR was obtained every 6 s. Eleven participants were excluded from HR analysis due to artifacts in the ECG. The SCR was recorded with two electrodes placed on the hypothenar eminence of the left hand. The SCR was measured in microSiemens and averaged every 6 s for each 2‐min block. Four participants were excluded from SCR analyses due to artifacts. To eliminate basal levels of HR and SCR, all epochs were transformed into difference scores by subtracting the average HR and SCR during the 3 s prior to each affective block from the within‐block measurements. Moreover, the first two epochs after starting each affective block were excluded from HR and SCR analyses to remove the 12‐s risetime of the pain stimulus.

**FIGURE 2:**
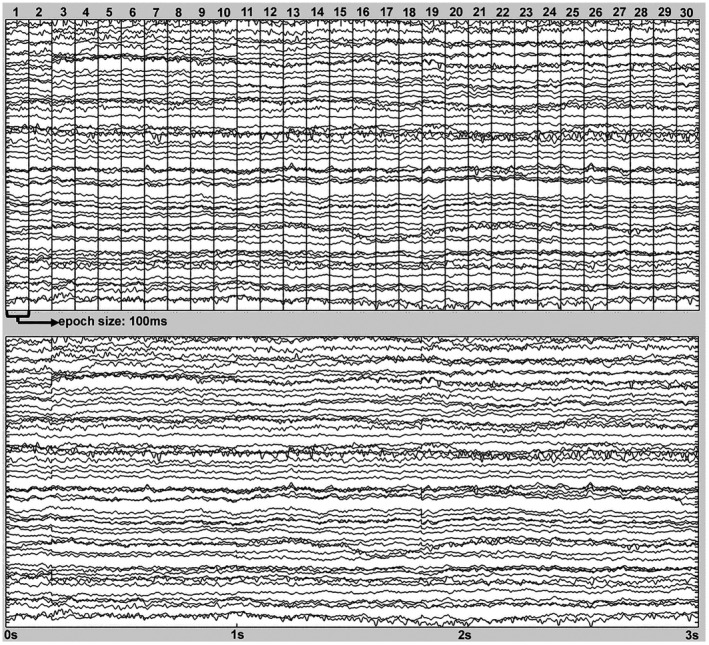
(a) Electroencephalogram preprocessed at 0.01 to 40 Hz and divided into epochs of 100 ms used in EEG functional connectivity analysis. (b) Continuous electroencephalogram after removing the epochs with artifacts used in EEG power analysis

The electroencephalogram was recorded from 58 electrodes with a vertex reference (Cz). Impedance was maintained at <50 kΩ. EEG preprocessing analysis was calculated with the EEGLAB toolbox of MATLAB (Delorme & Makeig, 2004). The electroencephalogram was rereferenced offline to the average of all electrodes and filtered with a 0.01‐Hz high‐pass filter and a 40‐Hz low‐pass filter. EEG waveforms were segmented into epochs of 100‐ms duration (1200 per block) with baseline correction to the 10 ms prior to each epoch. The selection of epoch size was based on the requirements to calculate the EEG functional connectivity measure for this study. The first 120 epochs after each affective block commenced were rejected to remove the risetime of the pain stimulus from the analyses. Then, epochs with amplitudes greater than ±70 μV were excluded, resulting in the retention of between 601 and 935 epochs per block. To standardize the number of epochs between blocks and participants, we randomly selected 601 epochs in all recordings (Figure 2a). Moreover, for EEG functional connectivity analysis, the signals were filtered in delta (0.5–4 Hz) and theta (4–8 Hz) frequency bands.

### 2.6 EEG power spectral analysis

EEG power was calculated in the remaining 1.1 min signal obtained after epoch exclusion (Figure 2b). Power spectral density was calculated for the 0.5–8 Hz interval of all channels at 0.5‐Hz resolution by using the Welch method (weighted overlapped segment averaging). The power data used in subsequent statistical analyses were log‐transformed and averaged in two frequency bands: delta (0.5–4 Hz) and theta (4–8 Hz). To reduce the number of comparisons in the statistical analyses, EEG channels were grouped into five regions: frontal (Fp1, AF3, F9, F7, F5, F3, F1, Fz, AFz, F2, F4, F6, F8, F10, AF4, and Fp2), central (FC5, FC3, FC1, C5, C3, C1, FCz, C2, C4, C6, FC2, FC4, and FC6), temporal (FT7, T9, T7, TP7, TP8, T8, T10, and FT8), parietal (CP5, CP1, P9, P7, P5, P3, P1, Pz, P2, P4, P6, P8, P10, CP2, and CP6), and occipital (PO3, O1, Oz, POz, O2, and PO4). Then, the average power of each frequency band was calculated for each EEG region.

### 2.7 EEG functional connectivity

The method of functional connectivity selected for our analysis was motif synchronization (MS). This method consists of counting the simultaneous appearance of predefined patterns or motifs between two time series (for further description of this method, see Rosário et al., 2015). Thus, the connectivity weight of each edge represents the number of times that a pair of electrodes was synchronized in a period of time. In other words, this index measures the similarity between two signals over time in relation to their amplitude fluctuations. MS was calculated with EEG signals filtered in delta and theta bands.

Functional connectivity was computed by using a sliding time window of 100 ms with 1‐ms steps. Thus, we obtained a synchronization matrix between pairs of electrodes (58 × 58 matrix) for each time window. To reduce spurious correlations, we shuffled all data points from the EEG signal to obtain an adjacent matrix and selected a threshold from the synchronization between the electrodes of the matrix. Our threshold was set at a 0.7 synchronization value, in which 70% of the motif fluctuation patterns from both electrode EEG signals were the same. This threshold was defined as the value at which chance synchrony was lower than or equal to 0.1%. Thus, synchronization values between electrodes that were greater than or equal to 0.7 were selected as significant from the correlation matrix, and the node degree was summed over time. The edge weight was normalized by dividing by the maximum edge weight value in the trial. To reduce the number of comparisons in the statistical analysis, weight node degrees were grouped into the same five regions described above (frontal, central, temporal, parietal, and occipital). Finally, we calculated the average edge weight for the frontocentral, frontoparietal, fronto‐occipital, centroparietal, and centro‐occipital regions, which are all related to emotional networks (Alipour et al., 2019; Güntekin et al., 2017; Lee et al., 2020; Miskovic & Schmidt, 2010; Wu et al., 2019).

### 2.8 Statistical analyses

Valence and arousal ratings were separately analyzed using a one‐way repeated‐measures analysis of variance (ANOVA), where EMOTION (pleasant, unpleasant, or neutral) was the single repeated‐measures factor. VAS scores were analyzed using a 3 × 2 repeated‐measures ANOVA using EMOTION (pleasant, unpleasant, or neutral) and PAIN (no pain/pain) as within‐subject factors.

Analyses of the SCR and HR in affective blocks consisted of a 3 × 2 repeated‐measures ANOVA using EMOTION (pleasant, unpleasant, or neutral) and PAIN (no pain/pain) as within‐subject factors.

Analyses of EEG power and functional connectivity were separately performed for delta and theta frequency bands. EEG power was analyzed with a 3 × 2 × 5 repeated‐measures ANOVA using EMOTION (pleasant, unpleasant, or neutral), PAIN (no pain/pain), and REGION (frontal, central, temporal, parietal, and occipital) as within‐subject factors. EEG functional connectivity was analyzed with a 3 × 2 × 5 repeated‐measures ANOVA using EMOTION (pleasant, unpleasant, or neutral), PAIN (no pain/pain), and REGION (frontocentral, frontoparietal, fronto‐occipital, centroparietal, and centro‐occipital) as within‐subject factors.

Sex differences were also examined in all analyses using sex (female or male) as a between‐subjects factor in the repeated‐measures ANOVAs. There were no significant sex differences, and this factor was removed from the final analyses.

In all analyses, the Greenhouse–Geisser epsilon correction was applied to adjust for the lack of sphericity. The results are reported with the original degrees of freedom and the corrected *p* values. When significant effects were found, post hoc analyses were performed using the Tukey correction. The level of significance was set at .050 for all analyses. Partial eta‐squared ($\eta_{p}^{2}$) was used as the effect size for *F* tests.

## 3 RESULTS

### 3.1 Subjective measures

**FIGURE 3:**
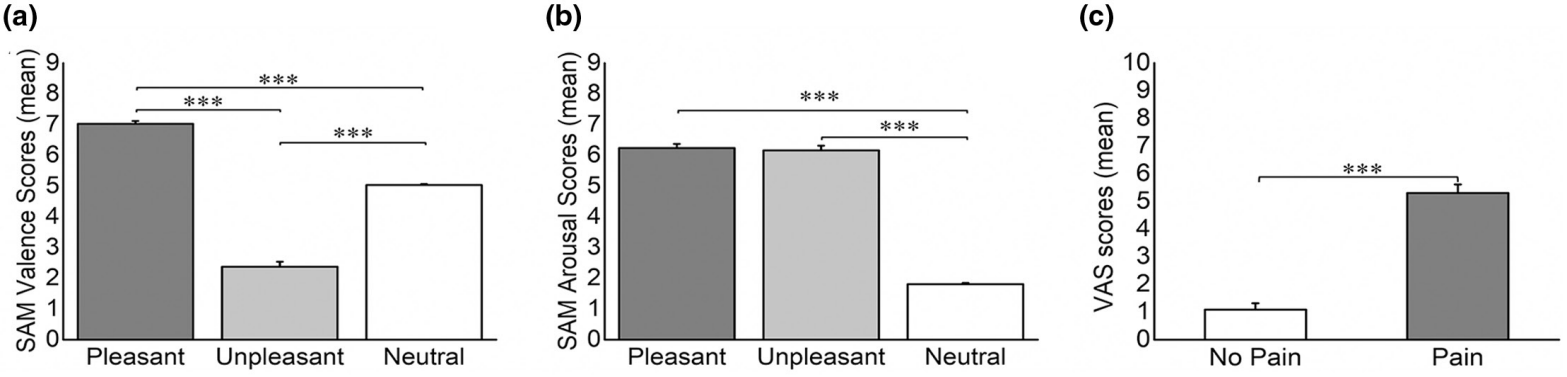
(a) Means of SAM valence scores for pleasant, unpleasant, and neutral images. (b) Means of SAM arousal scores for pleasant, unpleasant, and neutral images. (c) Means of pain unpleasantness ratings for no pain and pain stimulation. ***p < .001

Figure 3 presents the means and standard errors of the subjective response to affective images in terms of the valence and arousal ratings and the unpleasantness of temperature. One‐way ANOVAs revealed a significant effect of EMOTION for both the valence (*F*[2, 57] = 449.43, *p* < .001, $\eta_{p}^{2}$ = .94) and arousal (*F*[2, 57] = 461.92, *p* < .001, $\eta_{p}^{2}$ = .94) rating dimensions. Post hoc analyses indicated that valence ratings for pleasant images were higher than those for neutral and unpleasant images (both *p* < .001) and that valence ratings for unpleasant images were lower than those for neutral images (*p* < .001) (Figure 3a). Unpleasant and pleasant images were rated as more arousing than neutral images (*p* < .001) (Figure 3b).

The 3 × 2 repeated‐measures ANOVA on VAS scores revealed significant main effects of EMOTION (*F*[2, 74] = 3.54, *p* = .040, $\eta_{p}^{2}$ = .09) and PAIN (*F*[1, 37] = 191.78, *p* < .001, $\eta_{p}^{2}$ = .88). Post hoc analyses did not find significant differences between emotions, but they did reveal that the unpleasantness of temperature was higher during the pain condition than during the no‐pain condition (*p* < .001; Figure 3c). No significant EMOTION × PAIN interaction effect was found, suggesting that the unpleasantness of the pain stimulation was not affected by the affective content of the images.

### 3.2 Peripheral measures

**FIGURE 4:**
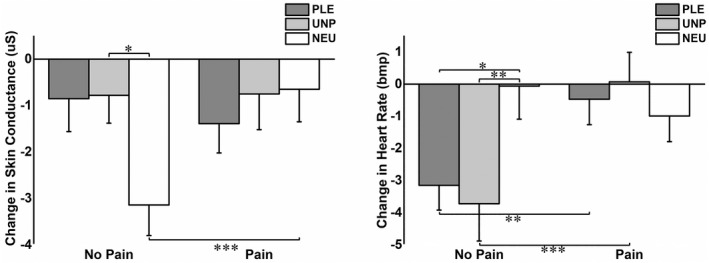
Mean and standard error of change in skin conductance and heart rate when viewing pleasant (PLE), unpleasant (UNP), and neutral (NEU) images during no‐pain and pain conditions. *p < .050, **p < .01, and ***p < .001

Figure 4 presents autonomic responses during pain and no pain stimulation conditions. The 3 × 2 repeated‐measures ANOVA on the SCR yielded a significant interaction effect of EMOTION × PAIN (*F*[2, 70] = 4.68, *p* = .012, $\eta_{p}^{2}$ = .12). Other effects were not significant. Post hoc tests of the EMOTION × PAIN interaction revealed that neutral/pain blocks evoked a greater SCR than neutral/no pain blocks (*p* = .001), suggesting that pain affected the processing of neutral images. No significant differences between pain conditions were found in other affective categories. Regarding each pain condition, a significant difference was found only between neutral/no pain blocks and unpleasant/no pain blocks (*p* = .038), and a nonsignificant trend was found between neutral/no pain blocks and pleasant/no pain blocks (*p* = .064). Thus, the lack of pain‐related SCR changes when viewing pleasant or unpleasant images could be due to a ceiling effect.

The 3 × 2 repeated‐measures (ANOVA) on HRs yielded a significant main effect of PAIN (*F*[1, 28] = 7.21, *p* = .012, $\eta_{p}^{2}$ = .21) and a significant EMOTION × PAIN interaction (*F*[2, 56] = 5.70, *p* = .007, $\eta_{p}^{2}$ = .17). The main effect of PAIN indicated that HR was increased in pain conditions compared with no‐pain conditions, regardless of emotional stimuli. Post hoc tests of the EMOTION × PAIN interaction revealed that neutral/no pain blocks evoked more pronounced HR acceleration than pleasant/no pain (*p* = .025) and unpleasant/no pain (*p* = .014) blocks. Furthermore, HR acceleration was more pronounced in pleasant/pain (*p* = .011) and unpleasant/pain (*p* = .004) conditions than their respective no‐pain conditions. In general, these post hoc analyses suggest that pain affected the affective processing of pleasant and unpleasant images and that the lack of pain‐related HR changes when viewing neutral images could be due to a ceiling effect.

### 3.3 EEG power

**FIGURE 5:**
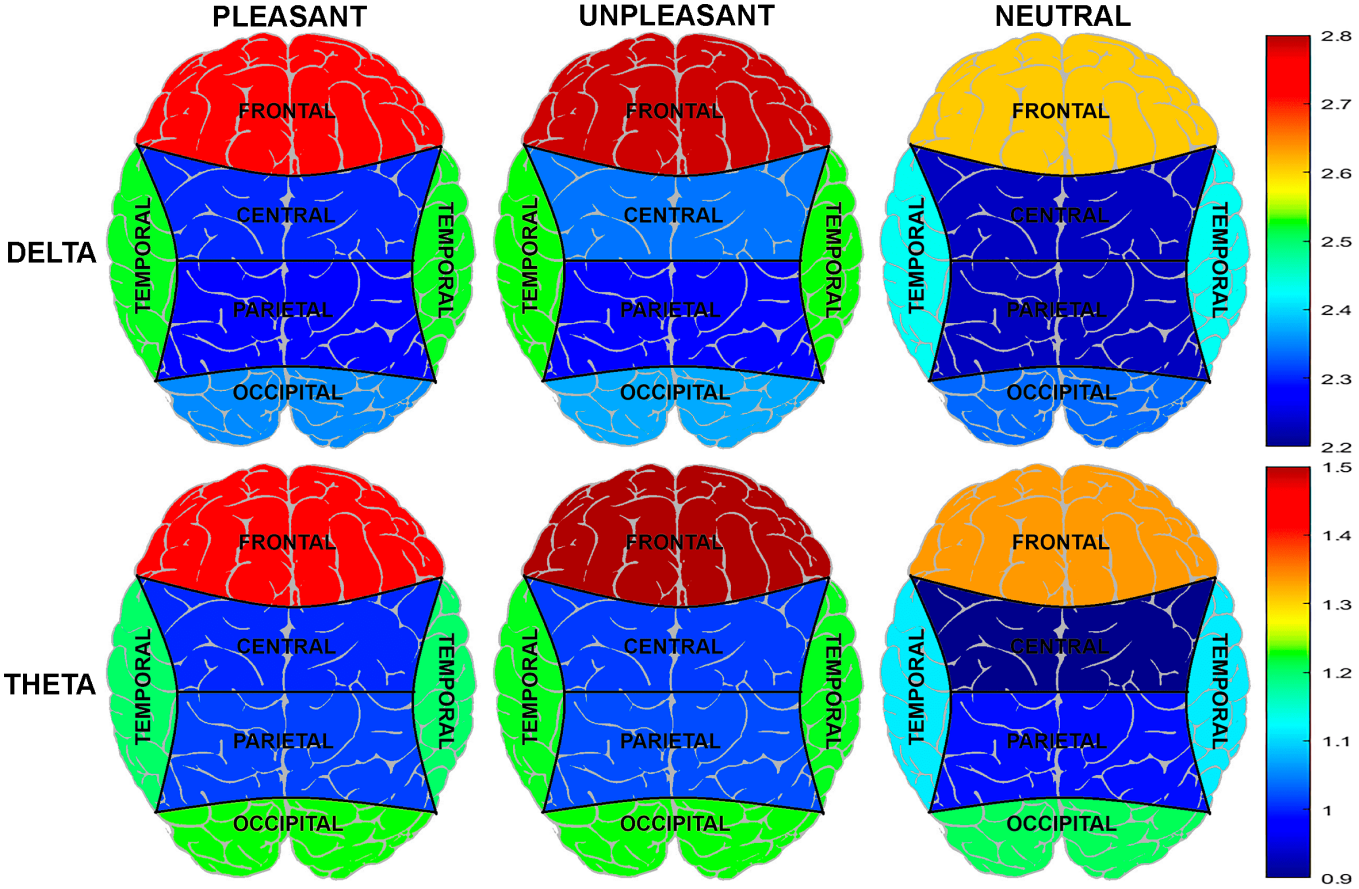
Means of delta and theta power for each affective block in frontal, central, temporal, parietal, and occipital regions

**FIGURE 6:**
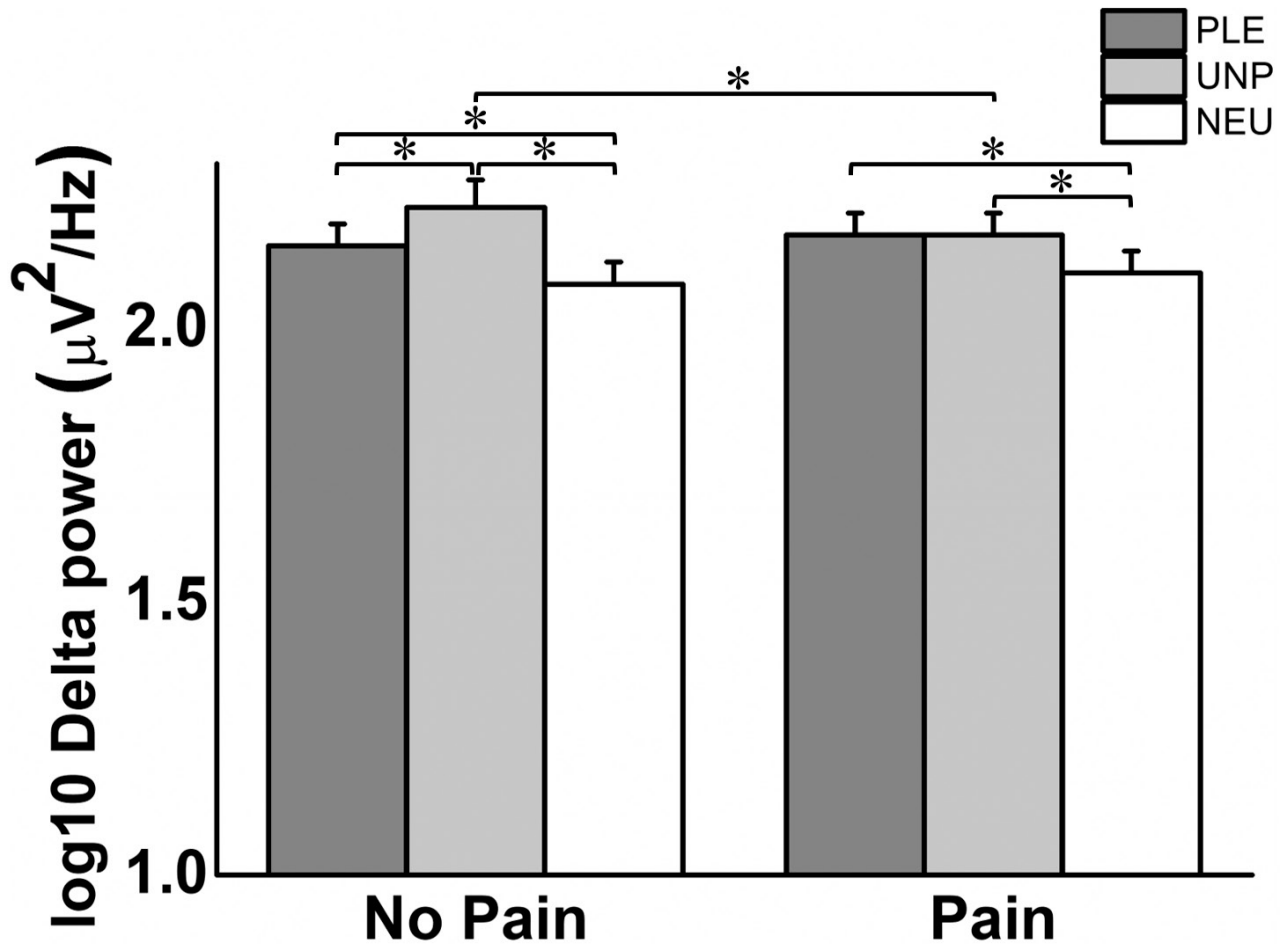
Means and standard errors of delta power in the central region in the no‐pain and pain conditions for each affective block. *p < .05

The 3 × 2 × 5 repeated‐measures ANOVA on EEG power revealed significant main effects of EMOTION and REGION as well as a significant EMOTION × REGION interaction on delta and theta frequency bands (Table 2). In contrast to our other findings, no effect on the power of these EEG frequencies due to PAIN were obtained. To further analyze the topographical distribution of these effects, additional 3 × 2 repeated‐measures ANOVAs were performed for the frontal, central, temporal, parietal, and occipital regions at each frequency band. Significant effects due to EMOTION were observed in the *delta* band in the following brain regions: frontal (*F*[2, 74] = 57.43, *p* < .001, $\eta_{p}^{2}$ = .61), central (*F*[2, 74] = 24.26, *p* < .001, $\eta_{p}^{2}$ = .40), temporal (*F*[2, 74] = 14.29, *p* < .001, $\eta_{p}^{2}$ = .28), and parietal (*F*[2, 74] = 4.20, *p* = .030, $\eta_{p}^{2}$ = .10). In all these brain regions, delta power was lower in response to neutral images than in response to pleasant and unpleasant images (Figure 5). Moreover, a significant EMOTION × PAIN interaction effect (*F*[2, 74] = 3.78, *p* = .028, $\eta_{p}^{2}$ = .09) was found in the central region. Post hoc pairwise mean comparisons revealed that *delta* power in response to unpleasant images was reduced during the pain condition compared with the no‐pain condition (*p* = .007), suggesting that pain affected the processing of unpleasant images. Pleasant/no pain (*p* = .009) and unpleasant/no pain (*p* < .001) blocks elicited higher delta power than neutral/no pain images. In addition, unpleasant/no pain (*p* < .001) blocks elicited higher delta power than pleasant/no pain blocks (Figure 6). Likewise, pleasant/pain (*p* = .002) and unpleasant/pain blocks (*p* = .006) elicited higher delta power than neutral/pain blocks.

**TABLE 2:** Significant results and effect sizes from the 3 × 2 × 5 ANOVA on EEG power in the delta and theta frequency bands

Frequency bands	EMOTION	REGION	EMOTION × REGION
Delta	*F*(2, 74) = 21.55*** $\eta_{p}^{2}$ = .37	*F*(4, 148) = 83.29*** $\eta_{p}^{2}$ = .69	*F*(8, 296) = 21.48*** $\eta_{p}^{2}$ = .37
Theta	*F*(2, 74) = 36.01*** $\eta_{p}^{2}$ = .49	*F*(4, 148) = 136.94*** $\eta_{p}^{2}$ = .79	*F*(8, 296) = 38.94*** $\eta_{p}^{2}$ = .51

** *p* < .01;

*** *p* < .001

Regarding *theta* power, significant effects due to EMOTION were found at the frontal (*F*[2, 74] = 70.80, *p* < .001, $\eta_{p}^{2}$ = .66), central (*F*[2, 74] = 33.18, *p* < .001, $\eta_{p}^{2}$ = .47), temporal (*F*[2, 74] = 42.16, *p* < .001, $\eta_{p}^{2}$ = .53), parietal (*F*[2, 74] = 5.59, *p* = .015, $\eta_{p}^{2}$ = .11), and occipital electrodes (*F*[2, 74] = 3.87, *p* = .028, $\eta_{p}^{2}$ = .10). In all these brain regions, theta power was lower in response to neutral images than to pleasant or unpleasant images (Figure 5).

### 3.4 EEG functional connectivity

**FIGURE 7:**
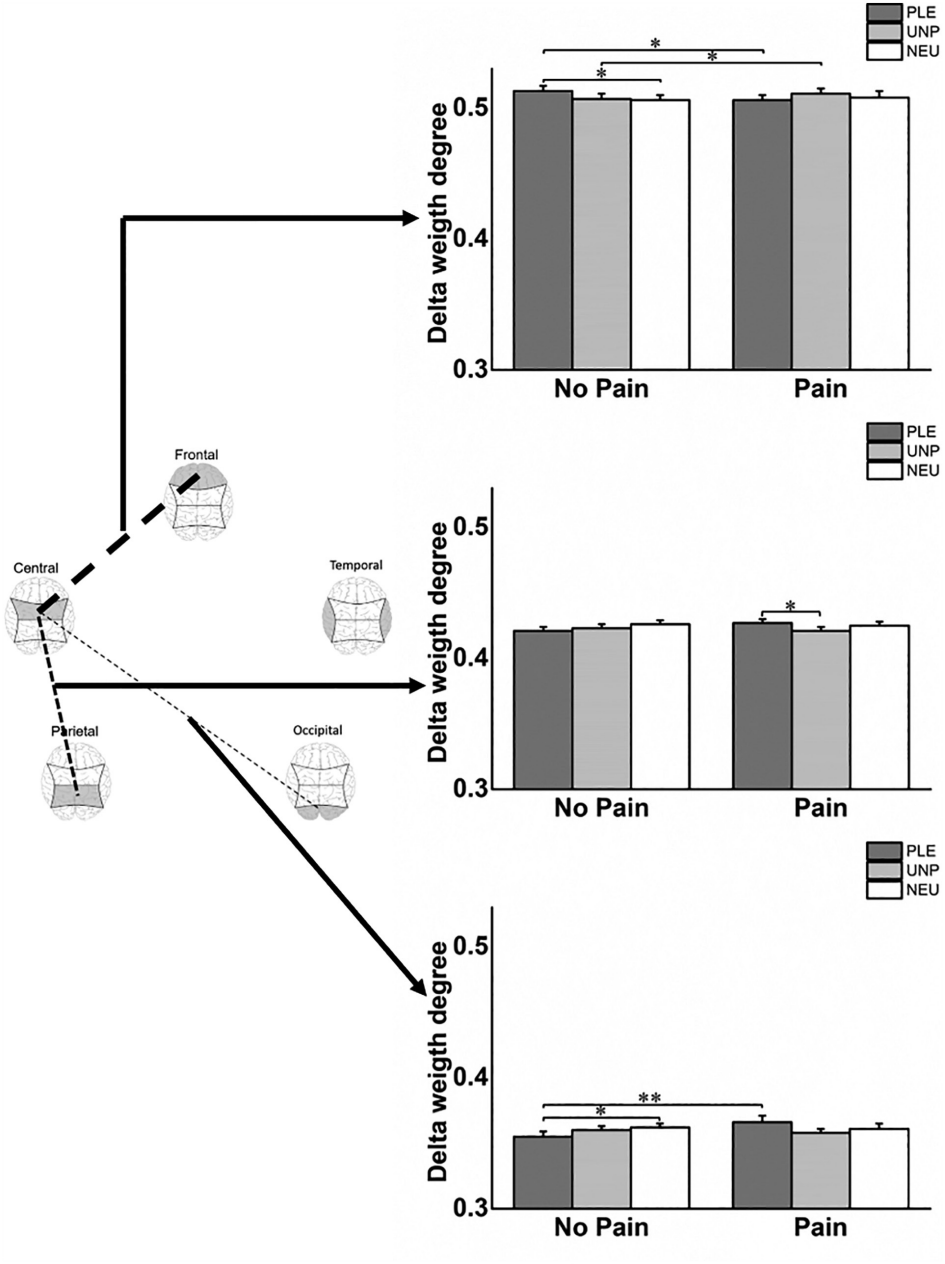
Means and standard errors of delta connectivity in no‐pain and pain conditions for each affective block. *p < .05 and **p < .01

A repeated‐measures ANOVA on the EEG functional connectivity data revealed a significant main effect of REGION (*F*[4148] = 775.50, *p* < .001, $\eta_{p}^{2}$ = .95) and a significant EMOTION × PAIN × REGION interaction (*F*[8296] = 3.62, *p* = .011, $\eta_{p}^{2}$ = .09) in the *delta* band. Post hoc pairwise mean comparisons revealed that pain decreased connectivity in frontocentral brain regions (*p* = .025) and to increased connectivity in centro‐occipital brain regions (*p* = .009) in response to pleasant images (Figure 7). In contrast, pain led to increased frontocentral connectivity in response to unpleasant images (*p* = .038). These results suggest that pain affected the delta EEG connectivity related to the processing of pleasant and unpleasant images. Furthermore, post hoc mean comparisons showed that the no‐pain condition displayed increased frontocentral connectivity in response to pleasant images compared with neutral images (*p* = .038) and displayed reduced centro‐occipital connectivity in response to pleasant images compared with neutral images (*p* = .036). In contrast, pain led to increased centro‐parietal (*p* = .042) connectivity in response to pleasant images compared with unpleasant images (Figure 7).

A repeated‐measures ANOVA on the EEG functional connectivity data revealed significant main effects of EMOTION (*F*[2, 74] = 6.84, *p* < .010, $\eta_{p}^{2}$ = .16), PAIN (*F*[1, 37] = 4.61, *p* < .050, $\eta_{p}^{2}$ = .11), and REGION (*F*[4148] = 847.16, *p* < .001, $\eta_{p}^{2}$ = .96) in the *theta* band. Post hoc mean comparisons indicated that pain, compared with no pain, resulted in overall increased functional connectivity.

## 4 DISCUSSION

The aim of the present study was to investigate how pain affects the attentional processing of emotional information. Galvanic skin conductance, heart rate and EEG power, and functional connectivity in delta and theta frequency bands were recorded while healthy participants viewed affective images (pleasant, unpleasant, and neutral) under two conditions: with pain and without pain. The participants reported higher levels of discomfort in the pain condition than in the no‐pain condition across all affective categories. Typical affective SCR and HR increases were observed when affective stimuli were presented in the no‐pain condition, but these peripheral changes disappeared when the pain was present. In accordance with the literature, our EEG data showed that affective stimuli elicited enhanced delta and theta responses compared with neutral stimuli. However, pain affected only delta frequency band responses in the central region during exposure to pleasant and unpleasant images. These results suggest that pain processing captures the brain’s attentional resources, thus damping emotional processing and affective responses.

Our main findings indicate that pain can modulate the delta power elicited by the processing of affective stimuli. Delta oscillations on central electrodes have previously been shown to be involved in attentional and decision‐making tasks, as well as in perception and emotional processing (Güntekin & Başar, 2016; Klados et al., 2009; Knyazev et al., 2009). As expected, high delta power at the central electrodes was related to high arousal images when the pain was not present (Balconi et al., 2009a, 2009b; Knyazev et al., 2009). The activity in the delta frequency band on central electrodes seems to be related to attention toward arousing stimuli (Balconi et al., 2009a, 2009b). Delta power on central electrodes when the pain was present decreased only in unpleasant image blocks. This result may support the theory that tonic pain is more arousing than unpleasant stimuli and thus decreased the attentional processing of this type of image. In contrast, subjective pain scores showed that the unpleasantness of temperatures was higher when the pain was delivered in the presence of pleasant, unpleasant, and neutral images. In accordance with previous studies (Wieser et al., 2012), pain subjective measures seemed to show that pain captures attention, regardless of the affective image category. Perhaps the lack of change in the delta power elicited by pleasant/pain and neutral/pain blocks could be interpreted as a grounding effect produced by pleasant and neutral images.

Previous research on neural correlates of affective processing has revealed that delta connectivity, especially of the frontal and central areas, may be related to attention toward arousing stimuli (Güntekin et al., 2017; Lee et al., 2020; Wu et al., 2019). Our findings showed that central delta connectivity was modulated specifically by viewing pleasant images when the the pain was not present. Frontocentral connectivity increased and centro‐occipital connectivity decreased when participants were exposed to pleasant images compared with neutral images when the pain was not present. Delta connectivity during unpleasant image exposure was situated in the middle, without significant differences in connectivity in either region when participants viewed pleasant and neutral images. Thus, frontocentral and centro‐occipital connectivity in the no‐pain condition seems to confirm that delta connectivity reflects attention to arousing stimuli.

When the pain was present, frontocentral delta connectivity decreased in response to pleasant images and increased in response to unpleasant images. Previous studies have suggested that changes in central delta connectivity upon the delivery of pain may reflect the exchange of painful sensory information between the somatosensory area and areas related to pain salience (Hu et al., 2013; Nani et al., 2019; Shen et al., 2019). Consistent with this idea, our frontocentral delta connectivity findings in pain conditions might reflect painful sensory information interfering with frontal attention toward emotional states. Thus, the decrease in frontocentral delta connectivity elicited by pain paired with pleasant images could reflect a decrease in the attentional processing of pleasant information (Godinho et al., 2008). Furthermore, the increase in frontocentral delta connectivity elicited by pain paired with unpleasant images could reflect an increase in the attentional processing of painful information (Rhudy et al., 2006, 2008; Roy et al., 2012; Williams & Rhudy, 2009, 2012). On the other hand, centro‐occipital delta connectivity increased in response to pleasant images when the pain was present. This finding might stem from the interference of painful sensory information with the visual processing of pleasant images, thereby blocking the attentional processing of these images (Godinho et al., 2008). In general, the delta connectivity results showed that painful stimuli might increase pain salience and decrease attention to affective stimuli in the presence of both pleasant and unpleasant images. In alignment with this, the analysis of subjective pain measures may confirm that when painful stimuli were delivered, attention toward pain information increased in the presence of both pleasant and unpleasant images.

As expected, the SCR and HR in the no‐pain condition followed the typical physiological pattern found with affective image exposure (Bradley, 2009; Wilson et al., 2020). More arousing stimuli evoked a larger SCR and more pronounced cardiac deceleration, whereas a modest SCR and less‐pronounced cardiac acceleration were evoked by less arousing stimuli. These physiological responses have been interpreted as favoring attention to motivational stimuli (Bradley, 2009). Thus, compared with neutral stimuli (mushrooms and household objects), stimuli with higher levels of activation (threat, mutilations, and erotic stimuli) are motivationally relevant stimuli that capture attention and prompt heightened orienting responses and increased information intake (Hajcak & Foti, 2020). When tonic pain was present, this physiological pattern disappeared; exposure to pleasant, unpleasant, and neutral images resulted in similar SCR and HR. The lack of differences between affective categories in the presence of tonic pain could indicate that painful stimuli blocked the attentional processing of emotional stimuli in favor of pain processing (Montoro et al., 2016; Montoya et al., 2005; Roa Romero et al., 2013). Along with our subjective pain measures and EEG results, this further supports the notion that tonic pain has enormous attentional capture abilities, which decrease the attentional processing of and emotional arousal from affective images (Wieser et al., 2012). Wieser et al. (2012) found that tonic pain captures attention and decreases physiological emotional responses regardless of the affective image category. In the current study, however, tonic pain affected peripheral emotional responses in different ways. The SCR increased only in response to neutral images, and HR accelerated only in response to pleasant and unpleasant images when the pain was present. These different effects of tonic pain on each affective response could have been due to a ceiling effect of the SCR to pleasant and unpleasant images and of the HR in response to neutral images when the pain was not present.

This is the first study to show that autonomic and EEG responses to affective stimuli can be modulated by tonic pain. In general, we observed that when the pain was delivered, peripheral and central changes in response to stimuli were enhanced, suggesting an involuntary demand for attention by pain and a concomitant reduction in affective processing. However, the present study did not measure pain salience and attention toward affective images. Replicating the present study with the addition of an affective attentional task, such as the visual search task of affective images (Ramírez et al., 2010), could help to separate the attentional and motivational contributions of tonic pain on affective visual processing. We assumed that pain captured attentional resources otherwise related to emotional processing because painful stimuli were more arousing than affective images. Only one pain intensity (60% pain tolerance) was used for each participant; replicating this study using different pain intensities could help to clarify whether the capture of affective attentional resources by pain is related to pain intensity. The effects of pain on emotional EEG responses were limited, which may have been due to the low spatial resolution of electroencephalography, as our recordings were unable to capture brain activity in subcortical regions. Studies using magnetic resonance imaging could shed light on how tonic pain affects subcortical emotional connectivity. In addition, statistical analyses were conducted using the means of physiological responses over time to reduce the number of comparisons and present the data parsimoniously because of the high number of analyses. It is well known that pain and physiological emotional responses are affected by temporal dynamics (Alazrai et al., 2017; May et al., 2019; Yang et al., 2020; Zhuang et al., 2017), and further analyses considering time as an independent variable should be performed. Additionally, our affective stimuli were limited to affective images. Future studies with other affective stimuli, such as music or film clips, that permit further analysis of attentional deficits that appear when these stimuli are processed in concurrence with painful stimuli are needed to study the effect of pain on affective processing in a natural context.

In summary, an increased galvanic skin response in the presence of neutral and painful stimuli and an acceleration in heart rate in the presence of affective and painful stimuli may suggest that tonic pain is a salient stimulus that captures attention, prompting heightened orienting responses and increased information intake. Central delta power and functional connectivity responses in the presence of tonic pain may suggest that pain‐related information successfully competes with affective stimuli for attentional resources. Our results are consistent with the idea that tonic pain reduces attentional processing to affective stimuli, given their importance for survival.

## ACKNOWLEDGMENT

This work was supported by the Spanish Ministry of Economy and Competitiveness (PSI2014‐57231‐R) and by the Andalusian Regional Ministry of Economic Transformation, Industry, Knowledge, and Universities (B‐SEJ‐028‐UGR18). Open Access Funding provided by Universidad de Granada.

## CONFLICT OF INTEREST

The authors declare that this research was conducted in the absence of any professional, commercial, or financial relationship that could be construed as a potential conflict of interest.

## AUTHOR CONTRIBUTIONS

**Guzmán Alba:** Data curation; formal analysis; investigation; methodology; software; writing – original draft. **Jaime Vila:** Supervision; writing – review and editing. **José G.V. Miranda:** Formal analysis; methodology; software; supervision. **Pedro Montoya:** Formal analysis; supervision; writing – review and editing. **Miguel A. Muñoz:** Conceptualization; formal analysis; funding acquisition; methodology; project administration; supervision; writing – review and editing.

## Supporting information


**TABLE S1** Normative ratings means and standard deviations (sd) of the IAPS images selected in the dimensions of valence and arousal
