# Supplementary material for: Tonic pain reduces autonomic responses and EEG functional connectivity elicited by affective stimuli
Source: Psychophysiology. 2022 Feb 6;59(7):e14018. doi: 10.1111/psyp.14018 (PMC9285073; doi:10.1111/psyp.14018)
Supplement: Supplementary file 1 — TABLE S1 Normative ratings means and standard deviations (sd) of the IAPS images selected in the dimensions of valence and arousal [file PSYP-59-0-s001.docx]

| Table S1. Normative ratings means and standard deviations (sd) of the IAPS images selected in the dimensions of valence and arousal. | | | | | |
| --- | --- | --- | --- | --- | --- |
| Set | Image | Valence_mean | Valence_sd | Arousal_mean | Arousal_sd |
| Negative | 1050 | 3.69 | 1.99 | 7.43 | 2.14 |
|  | 1113 | 3.77 | 2.07 | 6.77 | 2.02 |
|  | 2811 | 1.90 | 1.27 | 7.15 | 1.73 |
|  | 3064 | 1.47 | 1.28 | 7 | 2.72 |
|  | 3100 | 1.57 | 0.98 | 7.21 | 2.34 |
|  | 3170 | 1.54 | 1.01 | 7.61 | 1.93 |
|  | 3400 | 2.42 | 1.71 | 7.28 | 2.17 |
|  | 3550 | 2.17 | 1.24 | 7.33 | 1.81 |
|  | 6212 | 1.7 | 1.29 | 7.2 | 1.88 |
|  | 6250 | 3.14 | 2 | 7.21 | 1.86 |
|  | 6263 | 2.57 | 1.75 | 6.88 | 2.233 |
|  | 6313 | 1.94 | 1.4 | 7.26 | 2.12 |
|  | 6410 | 2.81 | 1.8 | 6.76 | 2.04 |
|  | 6550 | 1.8 | 1.26 | 7.99 | 1.64 |
|  | 6560 | 2.91 | 1.99 | 7.42 | 1.91 |
|  | 6570.1 | 2.45 | 1.67 | 6.85 | 2.23 |
|  | 9040 | 1.37 | 0.79 | 7.27 | 2.15 |
|  | 9120 | 2.21 | 1.62 | 6.78 | 2.08 |
|  | 9187 | 1.95 | 1.42 | 6.97 | 1.85 |
|  | 9400 | 1.78 | 1.33 | 7.01 | 2.14 |
| Positive | 4652 | 7.68 | 1.64 | 7.24 | 2.09 |
|  | 4658 | 7.11 | 1.64 | 7.44 | 1.73 |
|  | 4668 | 7.62 | 1.22 | 6.75 | 1.97 |
|  | 4669 | 7.32 | 1.57 | 7.21 | 1.75 |
|  | 4670 | 7.45 | 1.64 | 7.37 | 1.81 |
|  | 4672 | 7.31 | 1.59 | 7.04 | 1.82 |
|  | 4676 | 7.39 | 1.45 | 6.96 | 1.9 |
|  | 4681 | 6.46 | 1.97 | 6.88 | 1.5 |
|  | 8178 | 6.88 | 1.86 | 7.92 | 1.48 |
|  | 8185 | 7.4 | 1.53 | 7.15 | 1.9 |
|  | 8186 | 6.79 | 1.81 | 7.25 | 2.04 |
|  | 8193 | 6.34 | 1.61 | 6.76 | 1.89 |
|  | 8251 | 6.62 | 1.76 | 6.87 | 1.99 |
|  | 8300 | 6.63 | 2.24 | 7.07 | 2.08 |
|  | 8341 | 6.31 | 1.79 | 7.56 | 1.49 |
|  | 8370 | 7.33 | 1.57 | 7.34 | 1.81 |
|  | 8400 | 6.98 | 1.75 | 7.27 | 1.74 |
|  | 8490 | 7.88 | 1.55 | 7.39 | 1.92 |
|  | 8496 | 8.22 | 1.37 | 6.71 | 2.03 |
|  | 8499 | 7.87 | 1.44 | 7.16 | 1.85 |
| Neutral | 5530 | 5.57 | 1.63 | 3.17 | 2.08 |
|  | 5531 | 5.77 | 1.4 | 2.83 | 1.9 |
|  | 5532 | 5.52 | 1.48 | 3.08 | 2.04 |
|  | 5533 | 5.78 | 1.35 | 2.93 | 1.97 |
|  | 5534 | 5.46 | 1.44 | 3.11 | 1.96 |
|  | 7001 | 5.47 | 1.22 | 3.32 | 1.96 |
|  | 7002 | 5.62 | 1.15 | 2.33 | 1.82 |
|  | 7003 | 5.16 | 0.88 | 3.26 | 1.88 |
|  | 7004 | 5.08 | 1.13 | 3.13 | 1.98 |
|  | 7006 | 4.48 | 1.49 | 2.73 | 2.27 |
|  | 7009 | 5.41 | 1.18 | 2.25 | 1.79 |
|  | 7010 | 4.86 | 1.57 | 3.11 | 2.07 |
|  | 7012 | 5.09 | 1 | 3.26 | 2.02 |
|  | 7020 | 4.82 | 1.25 | 3.03 | 1.88 |
|  | 7025 | 5.04 | 1.2 | 2.35 | 1.77 |
|  | 7030 | 4.75 | 1.23 | 3.29 | 2.18 |
|  | 7031 | 4.64 | 1.28 | 2.94 | 1.88 |
|  | 7032 | 4.89 | 1.36 | 3.17 | 1.92 |
|  | 7035 | 5.07 | 1.43 | 3.19 | 2.16 |
|  | 7040 | 5 | 1.31 | 3 | 1.89 |
